# Biofilm Growth Causes Damage to Silicone Voice Prostheses in Patients after Surgical Treatment of Locally Advanced Laryngeal Cancer

**DOI:** 10.3390/pathogens9100793

**Published:** 2020-09-26

**Authors:** Jakub Spałek, Piotr Deptuła, Mateusz Cieśluk, Agnieszka Strzelecka, Dawid Łysik, Joanna Mystkowska, Tamara Daniluk, Grzegorz Król, Stanisław Góźdź, Robert Bucki, Bonita Durnaś, Sławomir Okła

**Affiliations:** 1 Institute of Medical Science, Collegium Medicum, Jan Kochanowski University of Kielce, IX Wieków Kielc 19A, 25-317 Kielce, Poland; jspalek@ujk.edu.pl (J.S.); g.krol@op.pl (G.K.); stanislaw.gozdz@onkol.kielce.pl (S.G.); bonita.durnas@onkol.kielce.pl (B.D.); 2 Department of Otolaryngology, Head and Neck Surgery, Holy-Cross Cancer Center, Artwińskiego 3, 25-734 Kielce, Poland; 3 Department of Medical Microbiology and Nanobiomedical Engineering, Medical University of Bialystok, Mickiewicza 2C, 15-222 Bialystok, Poland; piotr.deptula@umb.edu.pl (P.D.); mticv1@gmail.com (M.C.); tamara.daniluk@umb.edu.pl (T.D.); buckirobert@gmail.com (R.B.); 4 Institute of Health Science, Collegium Medicum, Jan Kochanowski University of Kielce, IX Wieków Kielc 19A, 25-317 Kielce, Poland; strzel@ujk.edu.pl; 5 Institute of Biomedical Engineering, Bialystok University of Technology, Wiejska 45C, 15-351 Bialystok, Poland; d.lysik@pb.edu.pl (D.Ł.); j.mystkowska@pb.edu.pl (J.M.)

**Keywords:** laryngeal cancer, voice prosthesis, biofilm, silicone

## Abstract

Voice prosthesis implantation with the creation of a tracheoesophageal fistula is the gold standard procedure for voice rehabilitation in patients after a total laryngectomy. All patients implanted with a voice prosthesis (VP) have biofilms of fungi and bacteria grow on their surface. Biofilm colonization is one of the main reasons for VP degradation that can lead to VP dysfunction, which increases the high risk of pneumonia. In a 20-month evaluation period, 129 cases of prostheses after replacement procedures were investigated. Microbiological examination of the biofilms revealed that there were four of the most common fungi species (*Candida* spp.) and a large variety of bacterial species present. We studied the relationship between the time of proper function of Provox VP, the microorganism composition of the biofilm present on it, and the degradation level of the silicone material. Evaluation of the surface of the removed VP using an atomic force microscope (AFM) has demonstrated that biofilm growth might drastically change the silicone’s mechanical properties. Changes in silicone stiffness and thermal properties might contribute to the failure of VP function. Our data can serve in future studies for the development of methods to prevent or inhibit biofilm formation on the VP surface that would translate to an increase in their durability and safety.

## 1. Introduction

**Figure 1:**
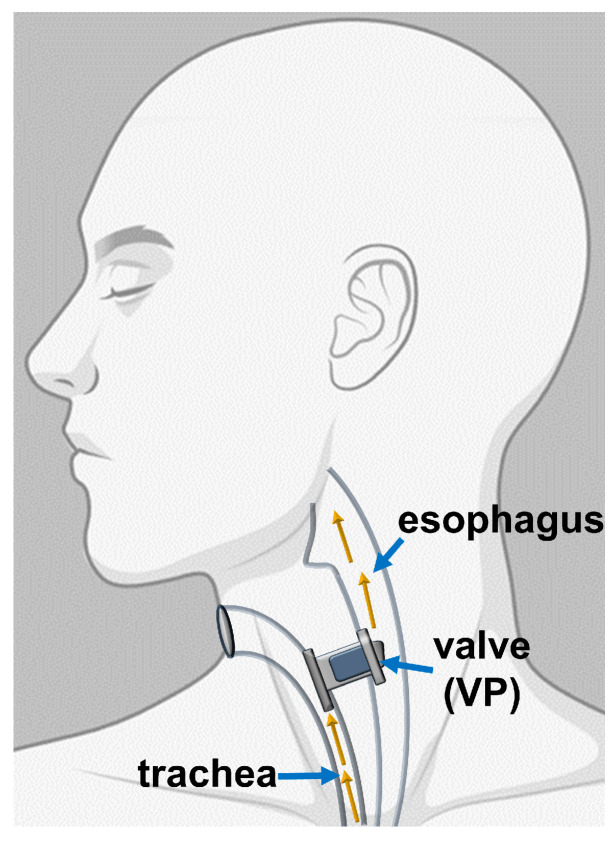
Schematic representation showing the location of implanted voice prosthesis (VP). The tracheoesophageal fistula is typically located on the posterior wall of the trachea, about 5 mm under the upper edge of the tracheostoma. There is a tightly placed VP inside the fistula that acts as a one-way valve.

In 2018, worldwide laryngeal cancer was diagnosed in 177,422 patients, and there were 94,771 deaths caused by it [1]. Total laryngectomy (TL) is a method of choice in the treatment of the advanced stages of laryngeal cancer (≥T3 in the TNM8 staging system) and as salvage treatment in the case of recurrence after primary organ preservation treatment. As a result of TL, a patient loses the physiological ability of speech and may experience social hardship. Vocal rehabilitation is essential to improve their quality of life [2]. The most effective method of voice rehabilitation among postlaryngectomy patients is tracheoesophageal (TE) puncture with voice prosthesis (VP) implantation [3]. The pioneer of this method was Polish professor Erwin Mozolewski, who published his results in 1972 [4]. Then in 1980, Singer and Blom developed a VP made of medical-grade silicone [5]. The indwelling low-resistance Provox voice prosthesis, developed in the Netherlands Cancer Institute in 1988, and the Blom–Singer VP are the most commonly used ones nowadays [6,7]. They both have a similar voice quality and lifetime [8]. The function of the TE fistula is to restore the connection between the airways (trachea) and the upper parts of the gastrointestinal tract (esophagus, pharynx, oral cavity) that has been lost due to TL. The mechanism of the implanted VP is that of a one-way valve to allow airflow from the trachea to the esophagus and to prevent esophageal content (mainly fluids) from leaking into the trachea (Figure 1).

The most serious disadvantage of silicone polymer-based voice prosthesis and other medical devices is their colonization by fungi and bacteria [9,10,11]. The main yeast strains isolated from the biofilms present on dysfunctional devices are *Candida albicans*, *Candida glabrata*, *Candida krusei*, and *Candida tropicalis*. On the VP surface, *Candida* spp. form a tridimensional network leading to prosthesis malfunction [12,13]. *Candida albicans* cells in the biofilm structure express the surface adhesion molecules Eap1 and Als3, which serve as specific surface receptors responsible for bacterial binding, including the viridans (oralis) groups *Streptococci* and *Staphylococcus aureus* [14,15]. In effect, mixed species bacteriofungal biofilm is the most common type of biofilm that develops on the silicone used to produce medical devices. The adherence of *Candida* species hyphae is crucial to initiating biofilm formation. The penetration of the yeast filaments is facilitated by the enzymatic degradation of silicone. The process of enzymatic silicone degradation might supply nutrients for growing yeast [12,16]. Moreover, free radicals (superoxide anion O^2−^) and extracellular enzymes released by monocytes and neutrophils (nicotinamide adenine dinucleotide phosphate oxidase (NADPH)) are also involved in the process of silicone damage [17]. Another reaction leading to silicone polymer degradation is oxidation caused by hydrogen peroxidase [18]. *Candida albicans* has 30 genes in which mutations could decrease adherence of hyphae. In addition to their genetic profile, the surface topography and host immunology are important factors controlling *Candida albicans* adherence [19,20]. Immune-competent cells present on the VP surface increase their migration upon activation by biofilm components [21]. However, the microorganisms residing within the biofilm structure are resistant to the host immune response and exogenous antimicrobial agents, mostly because they are protected by the biofilm’s extracellular matrix (ECM) [22]. Unlike the silicone polymer, the surrounding tissue is more susceptible to damage by immune cells. In some cases, voice prosthesis dysfunction is not only associated with valve blockage, but also with the destruction and deformation of the entire prosthesis and fistula inflammation. Fungal biofilms on medical devices are a reservoir of pathogens that can potentially lead to local or systemic infections [7,23]. However, in the context of VP, it is worth noting that except for local inflammatory reactions of tracheoesophageal fistula, there is no evidence to date of serious life-threatening infection caused directly by colonized microorganisms. On the other hand, it appears that the process of biofilm growth is the main reason for VP damage and deformation, which leads to its dysfunction. Central and lateral leakage of fluids by the tracheoesophageal fistula is the most common and potentially life-threatening dysfunction. It might lead to aspiration pneumonia [24,25,26]. The average lifetime of a single Provox voice prosthesis is about 4–6 months, but there are individual variations of the time of exploitation [27]. Factors influencing the time of voice prosthesis degradation are primary or postoperative irradiation for the head and neck region, as well as diet. Some studies have proven that consuming large quantities of dairy products has a significant effect on the extension of VP exploitation time [28]. 

Some attempts to elongate VP lifespan by reducing biofilm development include the use of nystatin, antimicrobial agents, oral wash, and to clean the VP by special brush. All have pros and cons but are still not fully satisfactory [29,30]. Looking for methods that will prevent initial adherence of hyphae (an essential step in biofilm formation) should be considered essential in the development of future VP material. 

This study has been conducted in part as a prospective cohort study and retrospective clinical study describing biofilms on Provox VPs removed from patients. We have identified four of the most common *Candida* species and a few groups of bacteria that should be considered as typical steps of VP colonization. Additionally, using scanning electron microscope (SEM) and atomic force microscope (AFM) analyses, we have characterized VP surface material damage caused by biofilm growth. Information on the qualitative composition of “microorganism inhabitants“ on VP and characteristics of the damage of the material that can be caused by biofilm growth provide a platform for further studies to develop new means to extend the time of VP exploitation and increase the safety of VP users after cancer treatment.

## 2. Results

### 2.1. Assessment of Microorganism Species Growing in Collected Biofilm Samples

On examined voice prostheses, we identified 13 different species of fungi (Table 1). The most common were *Candida krusei* (identified on 55.8% of 129 VPs), *Candida albicans* (46.5%), *Candida glabrata* (42.6%), *Candida tropicalis* (18.6%), and *Saccharomyces cerevisiae* (6.2%). We observed that there is no statistically significant difference between the occurrences of certain fungal species in each group (Table 1). Groups of patients were classified by the time of VP proper function, and they are fully characterized in Section 4.2. There is no relationship between fungal species growth and time of voice prosthesis exploitation.

There were many different species of bacteria identified on VPs from each group, and, for this study, most of them were included in three categories, as indicated in Table 2. *Pseudomonas aeruginosa* and *Staphylococcus aureus* have not been assigned to any of those categories and are shown separately, considering their high potential to cause local (tracheoesophageal fistula) or general (pneumonia) infections. The most common bacterial species was *Staphylococcus aureus* (44.2%). The second most commonly identified species were members that belong to a group of physiological oropharyngeal microbiota (32.6%). We have observed that there is no statistically significant difference between the prevalence of certain bacteria species in each group (Table 3). 

**Figure 2:**
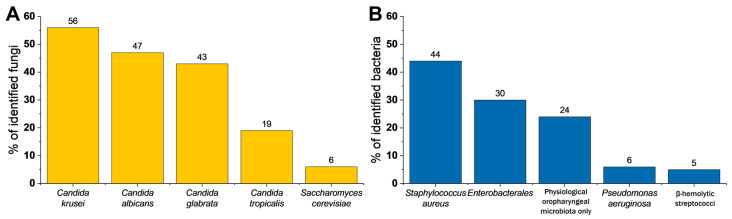
Total percentage of identified microorganisms in all exploitation time groups. (**A**) Percentage of identified fungi within the biofilm. (**B**) Percentage of identified bacteria within biofilms (*n* = 129).

Our study shows that for the most part, we identified a mix of bacterial and fungal species on the voice prostheses biofilms (83% of tested samples), but, in some cases, there were only bacteria (2.3%) or only fungi (14.7%) (Table 4). The occurrence of the most common species of fungi and bacteria categories identified on all voice prostheses is shown in Figure 2.

### 2.2. Microscopic Examination

**Figure 3:**
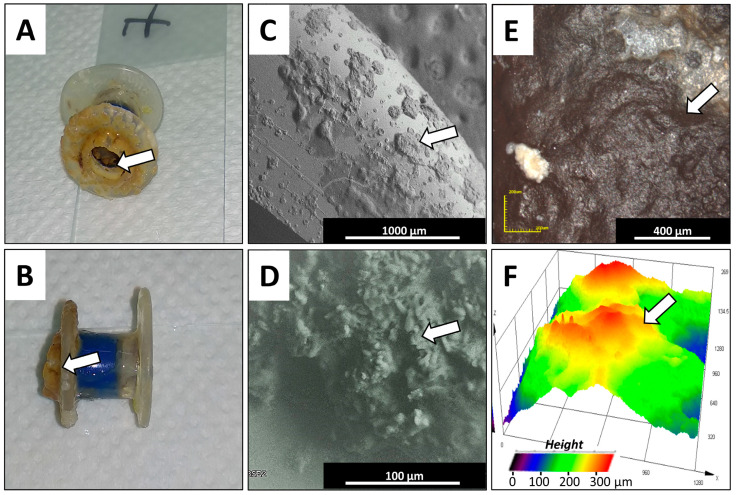
Macroscopic and microscopic views of the used silicone voice prosthesis from one representative VP collected from a patient from Group 5. Panels (**A**,**B**) show a general view of the used representative prosthesis, investigated using microscopic methods, with biofilm formations on the esophageal and tracheal flange surfaces and the prosthesis’ valve. Panels (**C**,**D**) show examples of the microscopic topography (SEM) from the representative prosthesis’ polymeric surfaces, showing biofilm formations. Panels (**E**,**F**) show the microscopic topography characterization (CLM microscope) of polymeric surfaces with biofilm formations, with 3D height visualization (white arrows show biofilm formations).

To evaluate the effects of biofilm growth on the properties of the silicone surface, collected VPs and attached biofilms were subjected to studies using SEM and a laser confocal microscope (CLSM). Prostheses from all groups that were described based on exploration time (Table 5) were investigated. The nature of surface changes was similar but more pronounced within Group 5. Figure 3 shows images of one representative (from the 10 tested) biofilm that formed on a VP removed from a patient who reported prosthesis dysfunction. Panels A and B represent morphological features of the esophageal flange and the valve, respectively. Macroscopic examination of the tracheal and esophageal parts of the removed prostheses showed adherent biofilm deposits that could be held responsible for the deterioration of the silicone rubber. Deposits of biofilm structures on both the valve and the internal ring of the valve suspension were observed. The esophageal surfaces of the investigated prosthesis—the esophageal flange, the valve flap, and the valve seating—were mostly covered by biofilm infestation. Observed colonization patterns ranged from single scattered biofilm deposits to those that fully cover the VP surface. Photos confirm that the flexible medical-grade silicone is especially prone to microbial infestation by biofilms. Panels C and D present SEM images of the esophageal flange with biofilm deposits. Detailed imaging of the adhered deposits revealed the characteristics of the biofilm on the polymer surface. Stiff and compact bulges in the biofilm layers on the flange were observed. Additionally, biofilm growth, with infiltration into silicone rubber, was observed. Panels E and F show a microscopic view (CLSM microscope) of the esophageal flange with biofilm covers. Panel F shows a 3D topography image with height measurements of the biofilm layer. The stiff biofilm deposition was observed to have a height of up to 0.5 mm. These dense and high biofilm deposits can impede valve closure and lead to transprosthetic leakage of esophageal contents into the trachea.

**Figure 4:**
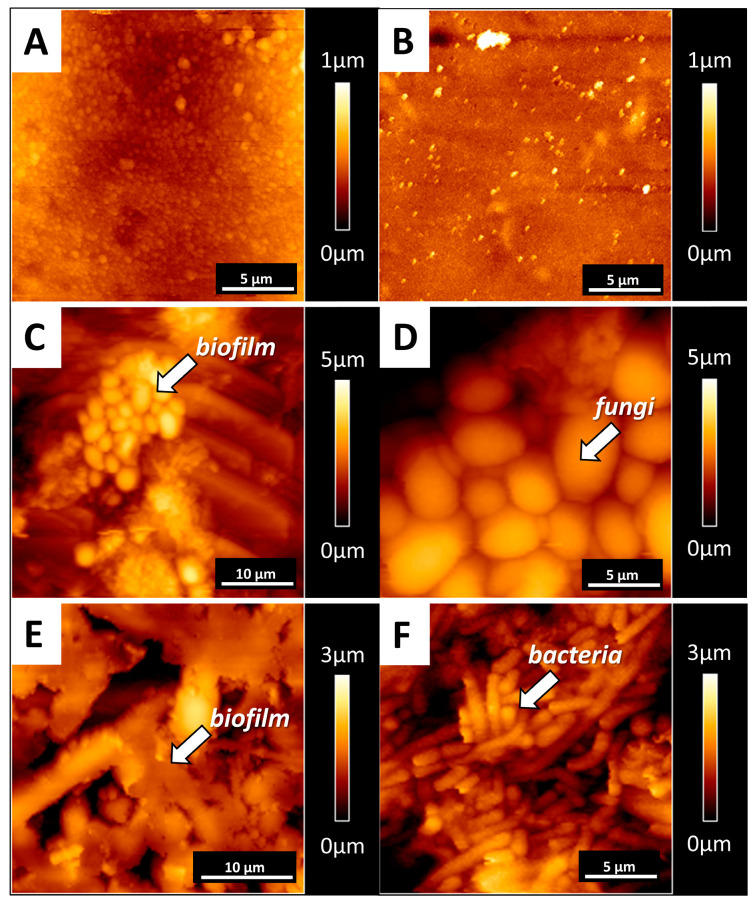
Microscopic (AFM) nanoscale views of the biofilm formed on the representative silicone voice prosthesis. Panels (**A**,**B**) show the topography of the control samples. Panels (**C**–**F**) show the polymer morphology, with formed biofilms where fungi or bacteria dominate. All images are representative of 10 different VPs removed from who reported prosthesis dysfunction and were included in Group 5 (*n* = 10).

Figure 4 shows the AFM morphology of biofilm deposited on the silicone surface. Panels A and B present the control—clear surfaces with remnants from manufacturing processes such as injection die molding. Panels C–F show biofilm structures. Detailed imaging of the adhered deposits revealed biofilm composition on the polymer surfaces. 

**Figure 5:**
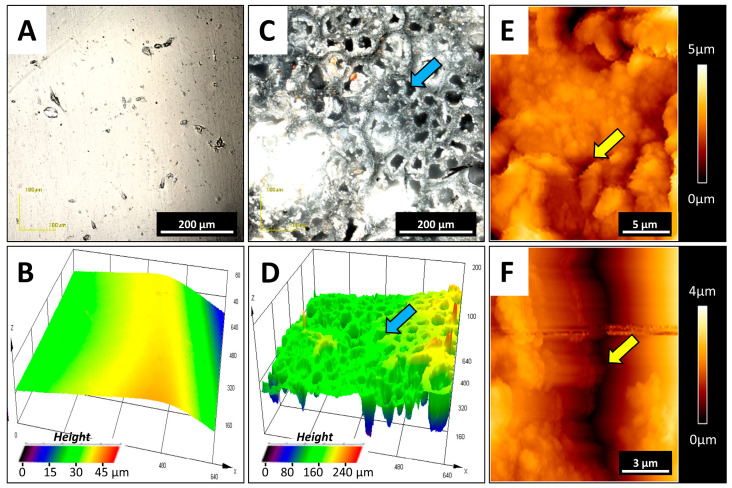
Polymer surface damage, visible after biofilm removal. Panels (**A**,**B**) show a picture and 3D topography of the control sample surfaces. Panels (**C**,**D**) show silicone surface porosity from the representative used prosthesis (blue arrows) as a result of interaction between tissue and silicone and biofilm action. Panels (**E**,**F**) show microcracks (yellow arrows) in the silicone surfaces. Panels (**A**–**D**) are from the CLM microscope; panels (**E**,**F**) are from the AFM microscope. All images are representative of 10 different VPs removed from patients who reported prosthesis dysfunction and were included in Group 5 (*n* = 10).

Figure 5 shows examples of silicone deterioration revealed after biofilm removal from the representative VP from the fifth time-group (*n* = 10). Panels A and B show control sample surfaces with remnants from the manufacturing process. Panels C–F show the topography of the silicone surface after biofilm removal using sonification. The interaction between tissue, material, and the development of biofilm might account for all material damage. Confocal microscopy revealed the porosity (blue arrows) of the silicone surface (Panels C and D). Microcracks (yellow arrows) were also visible using AFM (Panels E and F).

**Figure 6:**
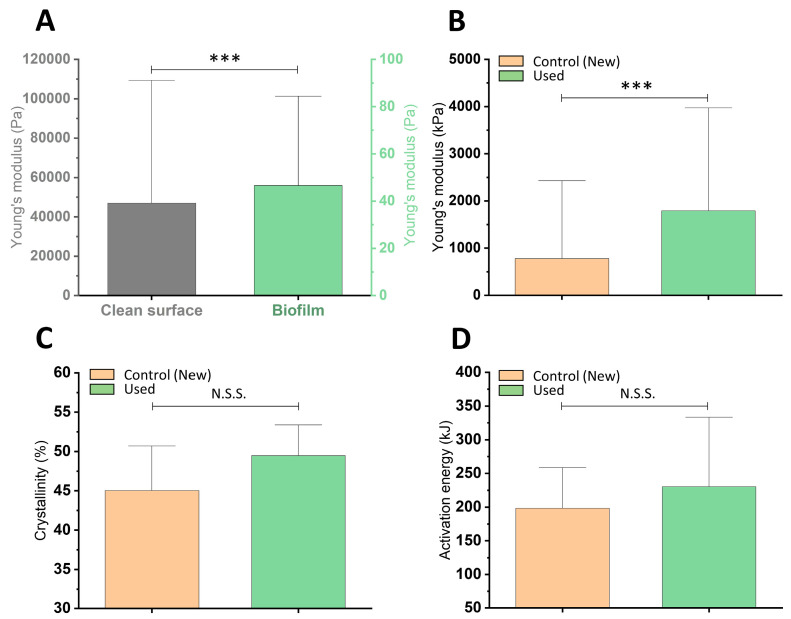
Mechanical and thermal properties of biofilm and silicone from representative VPs belonging to the fifth time-group. Panel (**A**) shows biofilm stiffness on the silicone surface compared to the clean material. Panel (**B**) shows changes in silicone surface stiffness after biofilm removal. Panels (**C**,**D**) show changes in the thermal properties of silicones under biofilm influence: changes in silicone crystallinity and silicone activation energy, respectively (*n* = 10, * *p* ≤ 0.05) were considered to be statistically significant; *** *p* ≤ 0.001; N.S.S.—not statistically significant).

Changes in the mechanical and thermal properties of the representative VP from the fifth group (*n* = 10) after biofilm removal are shown in Figure 6. Panel A indicates that the structures of the evenly developed biofilm are much less stiff than flexible medical silicone. Panel B shows the changes in Young’s modulus of the silicone. The biofilm–silicone interaction might result in a significant increase in polymer stiffness. The mean value of Young’s modulus of the control silicone was 780 kPa, while for the used silicone, it was 1800 kPa. Changes in the thermal parameters of the polymer, denoting internal changes in the structure, were also observed. Panels C and D show changes in the crystallinity and activation energy of the silicone prostheses. Mean values of crystallinity and activation energy of used VPs are higher compared to new VPs, but the differences between averages did not reach statistical significance. These changes were not as significant as in the case of Young’s modulus but determined a certain upward trend, and we can associate these changes with silicone mechanical changes. We observed not only the shape deformation of the prostheses, surface porosity, microcracks, and valve obstruction but also structural changes and degeneration.

## 3. Discussion

### 3.1. Analysis of the Study Group

The incidence of laryngeal cancer has a male-to-female ratio of 5:1 [31]. The higher ratio in our study (9:1) could be caused by the fact that the men were diagnosed at more advanced stages, by which time, the organ preservation treatment is not possible. Most of our patients had simultaneous implantation of voice prosthesis at the time of laryngectomy. The rest of them had undergone secondary implantation procedures similar to those in another study [32]. 

There is a variety of data published about VP lifespan. Some authors assessed that the average time of Provox Vega exploitation ranges from 3.5–18 months [27,28]. The average and median number of replacements per year is smaller than in another study, where the results were an average of 2.2 replacements and a median of 4.1 replacements per year [33]. There is a difference between our study and similar research, where patients were also categorized based on the period of exploitation. In the mentioned study, the most numerous group was the group of 4–6 months exploitation [32]. In our study, the longest time was 61 months. In this extreme case, the prosthesis was visibly covered by biofilm and was deformed, but it was still functioning well without any leakage. All analyzed patients did not have any symptoms of local or systemic infection. Moreover, none of our patients suffered from aspiration pneumonia. This observation agrees with previous reporting [32].

### 3.2. Assessment of Microorganism Species Growing in Collected Biofilm Samples

The variety of microorganisms identified during our study was large. The most common yeast identified on VPs were *Candida* species. Our results are similar to the results of other authors, but in our studies, the dominant species was *Candida krusei* instead of the previously reported *Candida albicans* [11,33]. In our study, similar to other publications, the most common bacterial species forming biofilms on VPs was *Staphylococcus aureus* [11,33]. Colonization of *Candida* spp. is highly associated with the destruction of the silicone material [16]. The biofilm growth, especially when taking place on the valve, can shorten the device’s lifetime. A biofilm containing a mixed flora of yeasts and bacteria, which we detect on the silicone surface, was similar to data reported in [11]. Interestingly, within the biofilm mass, it was possible to identify areas with either mostly bacterial or fungal cells. It has been suggested that initial adherence to the silicone polymer surface of *Candida* species can be preceded by bacteria. Fungi might create a conditioning biofilm on the soft surface of silicone prostheses. The presence of mixed microbial flora can be considered “a material coinfection”, with a synergistic interplay between bacterial and fungal cells [16]. 

However, the results have shown that there are no statistically significant correlations between the lifetime of voice prostheses and the microorganism composition of the biofilms that develop on the VPs. No species, neither yeasts nor bacteria, changed the lifespan of the voice prosthesis with statistical significance. 

We found that the most common yeasts identified on the VP biofilm belonged to *Candida* spp., but our statistical analysis has shown that there is no relationship between each of the species of *Candida* and the VP lifespan. We also found similar changes in the structural, thermal, and mechanical properties of the silicone material of the VP. Therefore, we concluded the VP’s degradation time and failure is not due to the presence of certain fungi or bacteria, but caused by the biofilm formation as a process, no matter the microbial composition. We assume that the structures of the biofilm are the decisive cause of valve nonclosure and the destruction of flanges through microcracks and material deformation [11,34]. Changes in the physicochemical properties during the exploitation when biofilm first develops on the VP surface may result from stress accumulation in some areas, leading to the polymer cracking. Cracks and deformation can cause the leaking of fluids through the prosthesis and failure of its function [11].

### 3.3. Microscopic Examination

As reported in [16,35], the esophageal surfaces of the investigated prosthesis–the esophageal flange, the valve flap, and the valve seating–were mostly covered by biofilm infestation. There were similar structures, like stiff and compact bulges in the biofilm layers, on the flanges described in previous studies [36,37,38,39] as we observed in our setting. Synthetic silicone is known to be rapidly colonized by microorganisms [11]. We confirmed that some silicone properties favor microbial colonization, and biofilm can cause the deterioration of polymer material. Prosthesis material can be not only superficially damaged but also penetrated, and their 3D structure can be disrupted by microorganisms. The deterioration of silicone surfaces is an interfacial process between the tissue–polymer surface and the polymer–biofilm surface and is related to the conditions prevailing on these surfaces. Overall, silicone deterioration in this setting can be considered a combination of biofilm formation [16], lytic processes, and extraction of soluble material compounds by microbial cells, and their products may lead to silicone embrittlement and structural damage [36]. Other studies suggest that the growth of biofilm generates an initial deterioration and long-term internal changes of the silicone through the adhesive forces of fungi and bacteria and the subsequent pressure increase, action of free radicals, and extracellular enzymes. Additionally, the presence of the biofilm activates the immune system and the production of proinflammatory molecules, which are involved in the mechanism of silicone damage [11,21]. The observed increase in crystallinity and activation energy of thermal decomposition of the polymers may indicate the effect of the biofilm on the length of polymer chains. Shortening of the chains can lead to easier crystalline rearrangement inside. Increased leaching of additives and monomers out of the polymer matrix, plasticizer degradation, and changes in crystallinity may all lead to elasticity changes and the increased embrittlement of the silicone [40].

The new polymeric material should be improved to prevent deformation and cracking during exploitation. A modification that will give us antimicrobial properties would also be highly desirable. One possibility to achieve such a goal is the addition of antimicrobial nanosystems or chemical compounds to polymers [41,42,43,44,45,46] that might reduce the ability of microorganisms to form and develop biofilms on the prosthesis’ surface.

It is worth underlining that patients involved in this study did not develop any systemic infection during the time the study was conducted. This observation suggests that microorganisms colonizing the VP are very unlikely to become the direct etiological factor of infections. However, it seems they lead to VP failure, which can cause leakage from the esophagus to the trachea, and it could be the main reason for aspiration pneumonia. Some previous publications have indicated that patients with a dysfunctional TE fistula had a three-fold higher risk of pneumonia than patients with the proper function of VP and TE fistula [47]. 

Even though the different factors that might govern biofilm formation on the VP surface are not yet defined, biofilm existence is a critical element from the perspective of the deterioration of VP silicone rubber. Additionally, the biofilm itself does not seem to be a hazard for the patient directly, but the consequences of voice prosthesis failure and dysfunction could lead to serious clinical complications.

## 4. Materials and Methods 

### 4.1. Voice Prostheses Collection 

We collected 187 dysfunctional Provox voice prostheses from 129 laryngectomized patients admitted to the Department of Otolaryngology, Head and Neck Surgery, of the Holy-Cross Oncology Centre in Kielce, Poland, over the span of 20 months. Some patients had replacement procedures more than once during that period—there were 58 such cases, but these prostheses were not included in the study. Before the replacement procedure, all patients were informed about the purpose and methods of the study. All patients agreed to participate in the study. The bioethical committee approved the study (Resolution of Bioethical Committee no. 16/2019). A physician, using sterile instruments, did the replacement procedure and collected the prostheses, which were placed into sterile containers. Directly after removal from the patients’ tracheoesophageal fistula, dysfunctional voice prostheses were subjected to microbiological evaluation. After the replacement procedure, patients answered questions about their medical history. Then, the information collected from the patients was compared with the data provided by the internal digital medical history system “CliniNet”. Correlations between the groups of collected prostheses and occurrences of certain fungi or bacteria species were calculated using the chi-square test of independence (variables such as the group of VP and the species of the microorganism were set as qualitative variables). 

### 4.2. Characteristics of the Study Group

Among all included patients, there were 116 males and 13 females. The male-to-female ratio was 9:1. The average age of patients was 66. The youngest patient was 47, and the oldest was 89. Patients underwent total laryngectomy from 18 years to 3 months (average 7.1 years) before the replacement procedure included in the study. Most of them (85%) had simultaneous implantation of voice prosthesis at the time of laryngectomy. The rest of them had undergone a secondary implantation procedure. In total, 88% of included patients had undergone radiation therapy: 73% as postoperative irradiation, and 15% as primary radical radiotherapy. The average number of voice prosthesis replacements per year in the studied patients after laryngectomy was 1.5 replacements (median 1.7), and the highest was 7 replacements. The average time between the previous replacement and the present replacement of voice prosthesis for a patient was 8.2 months (median time: 7 months).

**Figure 7:**
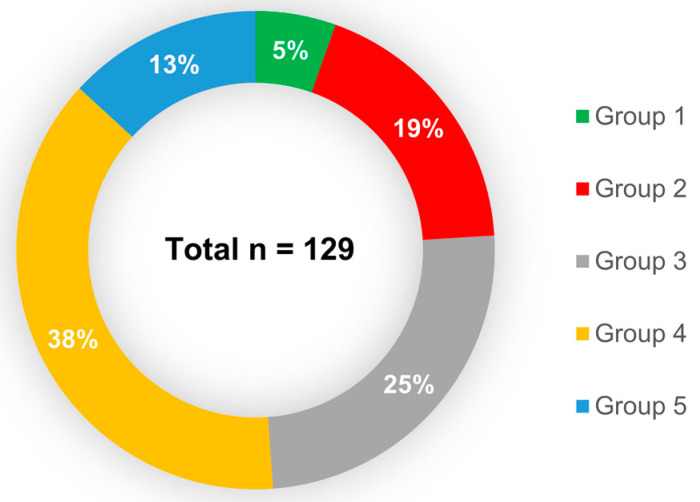
Proportion of the voice prosthesis included in each group, depending on the time of exploitation. Less than one month (Group 1), 1–3 months (Group 2), 4–6 months (Group 3), 7–12 months (Group 4), and over 12 months (Group 5). Total number of studied prostheses *n* = 129.

All patients and prostheses were categorized into five groups depending on the time of exploitation (Table 5). The most abundant was Group 4 (7–12 months of exploitation), with 49 collected prostheses. Group 1 (less than 1 month of exploitation) was the smallest in number (7 pcs of collected VPs; Figure 7). All patients used indwelling Provox voice prostheses (Table 6). The cause of replacement was categorized into two groups: device-related (63%) and fistula-related (37%). Device-related problems were mainly leakage through the valve and obstruction of the prosthesis, leading to increased airflow resistance during voicing. The fistula-related problems included leakage around the prosthesis, hypertrophy, and infection of tissues around the trachea–esophageal fistula. Accidental loss of prosthesis was not included in this study. In our study, there were no patients manifesting systemic infection that had been diagnosed in the time between the previous and the studied replacement procedures. Moreover, none of our patients had aspiration pneumonia during this time.

### 4.3. Assessment of Microorganisms Growing in Collected Biofilm Samples 

In recruited patients, directly after the VP replacement procedure, failed VPs were placed in sterile specimen containers and immediately transported to a microbiological laboratory. The voice prostheses were immersed in 5 mL of thioglycolate broth (Thermo Fisher Scientific, Waltham, MA, USA) and vortexed for 2–3 min. Then, 50 µL of the eluted material was seeded onto solid media for yeast and bacteria growth. The solid culture media used for microbiological examination was as follows: Sabouraud agar with and without antibiotics, Columbia agar with sheep’s blood, MacConkey agar, and hemophilus selective agar (all media were from Thermo Fisher Scientific). After a minimum of 48 h incubation, predominantly yeast and bacteria were identified using routine procedures that involved colony morphology examination and Gram staining. Then, identification of the species was performed using a Vitek 2 automated system (bioMerieux) with GNI, GPI, and Yeast ID cards.

### 4.4. Microscopic Studies

Macroscopic evaluation of the prostheses was performed within 5 h after removal. Biofilm morphology, coverage, and localization of plaques were assessed. Microscopic examination (topography) and mechanical measurements of the silicone prostheses and biofilm grown on their surfaces were recorded using a laser confocal microscope Olympus LEXT OLS4000 (CLSM), a scanning electron microscope Hitachi S-3000N (SEM), and an atomic force microscope JPK Instruments/Bruker NanoWizard 4 BioScience (AFM). SEM microscopy showed the biofilm characterization on the prosthesis surface. Images were made in BSE mode in a low vacuum. The CLSM microscope, in turn, made it possible to visualize the surface of the silicone after biofilm removal and observe their damage (3D topography maps). The topography of the biofilm on silicone surfaces was recorded using an AFM. Pyramidal-shaped cantilevers (OLYMPUS OMCL-RC800) were used and characterized by a spring constant of 0.1 N/m. Due to the lateral forces during contact mode scanning, a force-curve-based imaging mode was used (JPK QI™ mode—Quantitative Imaging). Topography maps of size 50 × 50 μm and 20 × 20 μm were carried out, with the resolution of 128 pixels per line, to show characteristics of the prosthesis surface and biofilm morphology. AFM experiments were done within 1 h of sample preparation, and silicone millimeter-scale pieces with biofilm were stored and kept in distilled water during experiments at room temperature. AFM ran in force-spectroscopy mode also served as a nanodetector for biofilm and polymer stiffness measurements. Elastic modulus (i.e., Young’s modulus) of the biofilm and polymers was calculated based on force-indentation curves. For biofilm stiffness measurements, force-indentation curves were collected on a stiff substrate and on the elastic biofilm parts. Due to the biofilm’s high deformability and complexity, force-indentation curves were collected using a silicone nitride cantilever with a spring constant of 2.5 N/m and a 20-μm diameter bead attached to the end. Elasticity maps with a scan area of 50 × 50 μm, corresponding to a grid of 2 × 2 pixels, were made in various places on the sample. By separation of the force curves recorded on the silicone surface and on the biofilm structures, we obtained the properties of the biofilm grown on prostheses. Local mechanical properties (Young’s modulus) of new and used prostheses were also calculated based on the force-indentation curves collected using OLYMPUS OMCL-RC800 cantilevers characterized by a spring constant of 0.4 N/m. Elasticity maps with a scan area of 20 × 20 μm, corresponding to a grid of 10 × 10 pixels, were made. All elasticity maps were collected from various sample areas in liquid conditions. Young’s moduli of biofilm and silicone were derived from the Hertz–Sneddon model applied to force-indentation curves [48] using the following formula:

$$F\left(\Delta z\right)=\frac{4}{3}\sqrt{R}\cdot E^{*}\cdot \Delta z^{1.5}$$

where $E^{*}$ is the apparent Young’s modulus:

$$\frac{1}{E^{*}}=\frac{1-\mu_{tip}^{2}}{E_{tip}}+\frac{1-\mu_{sample}^{2}}{E_{sample}}$$

If $E_{sample}$ ≪ $E_{tip}$ (as is true for living cells), then $\frac{1}{E^{*}}$ can be simplified:

$$\frac{1}{E^{*}}\approx \frac{1-\mu_{sample}^{2}}{E_{sample}}$$

$E_{sample}$ is Young’s modulus of the sample, and $\mu_{sample}$ is the Poisson ratio of the sample, related to the compressibility of the material [31,49] and assumed to be 0.5 for an incompressible material.

### 4.5. Thermal Measurements

Differential scanning calorimetry (DSC) studies were carried out using a DSC Discovery apparatus (TA Instruments, New Castle, DE, USA). The measurements were conducted in three cycles (heat–cool–heat) in a temperature range from −90 to 350 °C, with a heating rate of 10 °C/min and a cooling rate of 5 °C/min. The results discussed in the work were taken from the second heating curves (first heating and cooling were performed to reduce the thermal history of the tested samples). The obtained DSC curves were used to analyze melting temperature (Tm) and fusion enthalpy (∆Hm). The degree of the silicone’s crystallinity (Xc) was determined [50] according to the following equation:

$$X_{C}=\frac{\Delta H_{m}}{\Delta H_{m}^{100}}\cdot 100\%$$

where $\Delta H_{m}^{100}$ is the fusion enthalpy of 100% crystalline polydimethylsiloxane 38.2 J/g.

Thermogravimetry (TG) tests were carried out using a Q500 thermogravimetric analyzer (TA Instruments, New Castle, DE, USA) in a temperature range from 30 to 1000 °C and a nitrogen atmosphere, with heating rate (k) values of 5, 10, and 20 °C/min. Special attention was paid to the temperature at the start of thermal decomposition (*T*_DS_; taken as 1% of the weight loss of the sample). The Kissinger method was used to determine the activation energy of the thermal decomposition of the tested materials. This method is based on the dependence of the temperature value Tmax (corresponding to the maximum DTG signal) to the heating rate k:

$$\ln\left(\frac{k}{T_{max}^{2}}\right)=-\frac{E_{a}}{R}\cdot \frac{1}{T_{max}}+const.$$

As the heating rate increases, the temperature of the maximum intensity of the DTG signal also increases. The Kissinger method is based on presenting the obtained Tmax values in the configuration. The directional coefficient of the obtained straight line corresponds to the value (where Ea is the activation energy, and R is a gas constant equal to 8.31 J/mol·K).

### 4.6. Statistical Analysis

Statistical significance was determined using a two-tailed Student’s *t*-test for overall values. Statistical analyses were performed using OriginPro 9.65 (OriginLab Corporation, Northampton, MA, USA). *p*-values < 0.05 were considered to be statistically significant. Results are expressed as the average from all force curves for each group. Overall average values of Young’s modulus and thermal properties are presented as mean ± SD, where mean is the average value and SD is standard deviation. 

The lack of relationship between fungal or bacterial species occurrence and VP lifetime is presented as a result of the χ^2^ independence test in the column “*p*-value” of Table 1a and Table 3. The hypothesis of no correlation between bacterial or fungal species occurrence and time of VP exploitation (H0) vs. the hypothesis of the correlation between bacterial or fungal species occurrence and time of VP exploitation (H1 alternative) were tested. As the *p* test result is higher than the chosen significance level of α = 0.05, there is no reason to reject H0. Therefore, it can be concluded that the occurrence of a given species of fungus and bacteria is independent of the group of patients. For fungi and bacteria species, *p* > α, so we did not observe statistical significance.

## 5. Conclusions

The formation of biofilms and the subsequent material deterioration by fungal and bacterial growth are the main reasons for VP failure in laryngectomized patients. Changes in the mechanical and thermal properties of silicone by the process of biofilm formation were also observed. The most common fungal species on the VPs were *Candida krusei*, *Candida albicans, Candida glabrata*, and *Candida tropicalis*. The most common bacterial species was *Staphylococcus aureus*. We have observed that there is no statistically significant difference between the occurrence of certain fungal or bacterial species and the time of VP function upon implantation. No patients involved in this study developed any systemic infection that could be associated with microorganisms residing in VP biofilm. New polymeric material that is developed to produce VPs should be improved to prevent deformation and cracking during exploitation.

## Figures and Tables

**Table:** (**a**)

Species/Group of the Prostheses	Group 1 (*n* = 7) *n* (%)	Group 2 (*n* = 24) *n* (%)	Group 3 (*n* = 32) *n* (%)	Group 4 (*n* = 49) *n* (%)	Group 5 (*n* = 17) *n* (%)	Total (*n* = 129) *n* (%)	*p* = Value
*Candida krusei*	3 (2.3)	13 (10.1)	16 (12.4)	28 (21.7)	12 (9.3)	72 (55.8)	*p =* 0.648 *
*Candida albicans*	3 (2.3)	11 (8.5)	16 (12.4)	24 (18.6)	6 (4.7)	60 (46.5)	*p =* 0.882 *
*Candida glabrata*	4 (3.1)	9 (7.0)	11 (8.5)	20 (15.5)	11 (8.5)	55 (42.6)	*p =* 0.268 *
*Candida tropicalis*	2 (1.55)	4 (3.1)	7 (5.4)	9 (7.0)	2 (1.55)	24 (18.6)	*p =* 0.865 *
*Saccharomyces cerevisiae*	0 (0.0)	1 (0.8)	1 (0.8)	5 (3.9)	1 (0.8)	8 (6.2)	*p =* 0.581 *

* χ^2^ test; *p* > 0.05.

**Table:** (**b**)

Species/Group of the Prosthesis	Group 1 (*n* = 7) *n*	Group 2 (*n* = 24) *n*	Group 3 (*n* = 32) *n*	Group 4 (*n* = 49) *n*	Group 5 (*n* = 17) *n*	Total (*n* = 129) *n*
*Candida dubliniensis*	0	0	1	0	0	1
*Candida kefyr*	0	1	1	2	0	4
*Candida lusitaniae*	1	0	0	0	0	1
*Cryptococcus neoformans*	0	0	1	0	0	1
*Candida norvegensis*	0	0	1	2	0	1
*Candida parapsilosis*	0	1	0	1	0	2
*Rhodotorula glutinis*	0	0	2	1	1	4
*Cryptococcus laurenti*	0	0	0	1	0	1
No species identified	0	1	0	3	0	4

**Table 2:** Bacteria identified on VPs. When possible, each individual species was classified into wider groups for the purpose of this study—see column no. 1 “Category” (Cat.).

Cat.	Species	Group 1 (*n* = 7) *n*	Group 2 (*n* = 24) *n*	Group 3 (*n* = 32) *n*	Group 4 (*n* = 49) *n*	Group 5 (*n* = 17) *n*	Total (*n* = 129) *n*
I *	*Citrobacter freundi*	0	1	1	0	0	2
	*Enterobacter cloacae*	0	0	1	0	0	1
	*Escherichia coli*	0	1	3	2	3	9
	*Klebsiella oxytoca*	0	1	0	4	1	6
	*Klebsiella pneumoniae*	1	1	2	1	1	6
	*Morganella morganii*	0	0	0	1	0	1
	*Proteus hauseri*	0	0	0	0	1	1
	*Proteus mirabilis*	0	0	2	3	2	7
	*Raoultella planticola*	0	1	1	2	0	4
	*Serratia marcescens*	0	2	3	1	0	6
II *	*Streptococcus agalactiae*	1	1	3	1	0	6
	*Streptococcus* group C	0	0	1	0	0	1
III *	*Streptococcus* spp.	2	4	6	5	0	17
	*Streptococcus* viridans	0	1	0	5	0	6
	*Enterococcus* spp.	0	0	0	0	1	1
	*Staphylococcus* coag. (−)	0	2	1	3	0	6
No Cat. *	*Pseudomonas aeruginosa*	1	2	3	1	1	8
	*Staphylococcus aureus*	3	11	16	23	4	57

* I—enterobacteria; II—β-hemolytic streptococci; III—physiological oropharyngeal microbiota; No Cat.—not categorized.

**Table 3:** An overview of the bacteria identified on the investigated voice prostheses. Statistical analysis of the main groups of bacteria species in relation to all prostheses (1–5).

Species/Group of the Prosthesis	Group 1 (*n* = 7) *n*	Group 2 (*n* = 24) *n*	Group 3 (*n* = 32) *n*	Group 4 (*n* = 49) *n*	Group 5 (*n* = 17) *n*	Total (*n* = 129) *n*	*p* = Value
*Staphylococcus aureus*							
	3 (2.3)	11 (8.5)	16 (12.4)	23 (17.8)	4 (3.1)	57 (44.2)	*p =* 0.439 *
Physiological oropharyngeal microbiota							
	2 (1.55)	13 (10.1)	10 (7.75)	14 (10.85)	3 (2.3)	42 (32.6)	*p =* 0.131 *
Enterobacteria							
	1 (0.78)	6 (4.65)	12 (9.3)	14 (10.85)	6 (4.65)	39 (30.2)	*p =* 0.675 *
Physiological oropharyngeal microbiota only							
	1 (0.78)	11 (8.5)	6 (4.65)	11 (8.5)	2 (1.55)	31 (24.0)	*p =* 0.090 *
ß-hemolytic streptococci							
	1 (0.78)	1 (0.78)	4 (3.1)	1 (0.78)	0 (0.0)	7 (5.4)	*p =* 0.166 *
*Pseudomonas aeruginosa*							
	1 (0.78)	2 (1.55)	3 (2.3)	1 (0.78)	1 (0.78)	8 (6.2)	*p =* 0.527 *

* χ^2^ test; *p* > 0.05.

**Table 4:** An overview of the nature of biofilm present on the investigated voice prostheses. Statistical analysis of the main groups of bacteria species identified in relation to all prostheses (1–5).

Type of Biofilm	Occurrence of the Certain Type of Biofilm in Each Group (%)					
	Group 1	Group 2	Group 3	Group 4	Group 5	Total
mixed (bacteria/fungi)	71.4	87.5	93.8	79.6	70.6	83.0
fungi	28.6	8.3	6.3	16.3	29.4	14.7
bacteria	0.0	4.2	0.0	4.1	0.0	2.3

**Table 5:** Number of tested voice prostheses categorized by the time of exploitation and the sex of the patient. Abundance and characteristics of each group are listed in the table. Total number of collected prostheses *n* = 129.

The Time of Exploitation	Number of Prostheses Tested *n* (%)	Number of Males *n* (%)	Number of Females *n* (%)
Total	129 (100)	116 (90)	13 (10)
<one month (Group 1)	7 (5)	6 (4.7)	1 (0.8)
1–3 months (Group 2)	24 (19)	21 (16.3)	3 (2.3)
4–6 months (Group 3)	31 (25)	28 (21.8)	4 (3.1)
7–12 months (Group 4)	49 (38)	47 (36.4)	2 (1.6)
*>*12 months (Group 5)	17 (13)	14 (10.9)	3 (2.3)

**Table 6:** Type and size of collected voice prostheses. All collected voice prostheses were Provox. There were different types (standard Provox Vega, standard Provox 2, and XS-extra seal) and sizes. In the table are the presented numbers of each voice prosthesis by type and size.

Type of Voice Prosthesis	Number of Prostheses Tested *n* (%)
Total	129 (100)
Provox Vega	119 (92)
4 mm	1
6 mm	30
8 mm	66
10 mm	17
12.5 mm	5
Provox 2	4 (3)
Provox XS	6 (5)

## Abbreviations

VP	Voice prostheses
TL	Total laryngectomy
SEM	Scanning electron microscope
AFM	Atomic force microscope
TE	Tracheoesophageal
ECM	Extracelullar matrix
NADPH	Nicotinamide adenine dinucleotide phosphate
CLSM	Laser confocal microscope
DSC	Differential scanning calorimetry
TG	Thermogravimetry
